# IgLON5 autoimmunity in a patient with Creutzfeldt–Jakob disease: case report and review of literature

**DOI:** 10.3389/fneur.2024.1367361

**Published:** 2024-03-20

**Authors:** Xiaofeng Li, Yimin Chen, Le Zhang, Wei Zhang, Bin Li, José Fidel Baizabal-Carvallo, Xingwang Song

**Affiliations:** ^1^Department of Neurology, The Second Affiliated Hospital of Guangzhou Medical University, Guangzhou, China; ^2^Department of Neurology, Foshan Sanshui District People’s Hospital, Foshan, Guangdong, China; ^3^Department of Sciences and Engineering, University of Guanajuato, León, Mexico

**Keywords:** Creutzfeldt–Jakob disease, IgLON5 antibodies, autoimmune encephalitis, neurodegenerative disease, rapid progressive dementia

## Abstract

**Objective:**

We present the case of a patient with clinical and imaging features of sporadic Creutzfeldt–Jakob disease (sCJD) and positive IgLON5 antibodies (Abs) in the serum and CSF.

**Case report:**

A 66-year-old Chinese man presented to the hospital with a stroke-like episode, followed by rapidly progressive cognitive decline, mutism, and parkinsonism. The MRI results showed a cortical ribboning sign in diffusion-weighted MRI, periodic triphasic waves with a slow background in EEG, and positive protein 14–3-3 in CSF. There were matching IgLON5 Abs in the serum and CSF. A literature review showed positive autoimmune encephalitis Abs or autoimmune inflammatory disease between 0.5 and 8.6% among patients with clinical suspicion of CJD, most commonly anti-voltage-gated potassium channel (VGKC) complex and anti-N-methyl-D-aspartate receptor (NMDAR) Abs; however, IgLON5 autoimmunity in CJD has been rarely reported. This is an intriguing association as both conditions have been associated with brain deposits of phosphorylated tau protein.

**Conclusion:**

IgLON5 Abs may be observed in patients with a diagnosis of CJD; it is unknown whether a synergistic effect of IgLON5 Abs with CJD exists, increasing neurodegenerative changes.

## Introduction

Creutzfeldt–Jakob disease (CJD) is a fatal and potentially transmissible neurodegenerative disease caused by misfolded prion proteins (PrP^Sc^) (1). CJD was first described in 1920, and currently, four etiologies have been identified, namely, sporadic, variant, genetic/familial, and iatrogenic (2). CJD presents with rapidly progressive cognitive deterioration usually combined with myoclonus, cerebellar signs, tremor, spasticity, parkinsonism, mutism, and visual disturbances (1). The sporadic (sCJD) form is the most common etiology, representing 85–95% of all cases of CJD (2).

CJD is an important consideration when clinicians evaluate patients with rapid progressive dementia. Among those patients, autoimmune encephalitis (AE), particularly IgLON5 encephalitis, has recently emerged as an important differential diagnosis (3). IgLON5 is a neuronal molecule implicated in cell adhesion, neuronogenesis, and neuroplasticity (3). IgLON5 encephalitis is an antibody-mediated disorder characterized by abnormal sleep movements and behaviors, obstructive sleep apnea, bulbar symptoms, gait instability, supranuclear gaze palsy, movement disorders, and cognitive dysfunction (4). Other manifestations include oculomotor abnormalities, dysautonomia, neuropsychiatric symptoms, and motor neuron disease-like syndrome (4). The rich clinical phenomenology of IgLON5 autoimmunity overlaps with CJD manifestations.

In this report, we present a case diagnosed with sCJD and IgLON5 antibodies and analyze the possible relationship between both disorders. Additionally, we report on previous studies assessing cases with positive neuronal surface antigen (NSA) antibodies (Abs) in patients with suspected CJD.

## Case

**Figure 1:**
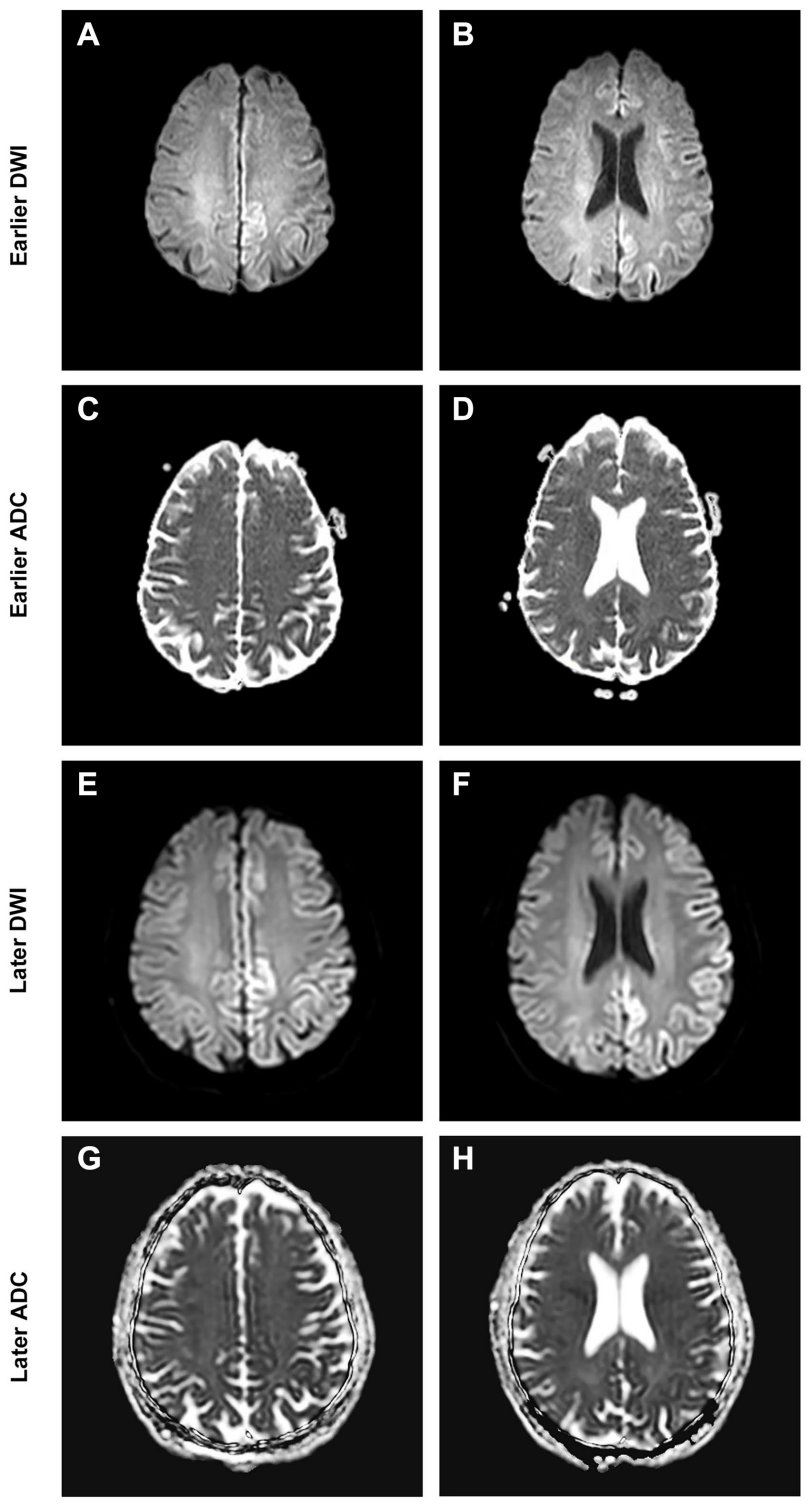
Brain MRI at presentation shows cortical DWI hyperintensities “cortical ribboning” **(A,B)** with ADC hypointensity **(C,D)** over the corresponding cortex. Brain MRI at follow-up shows spreading of the cortical restriction in DWI **(E,F)** and ADC **(G,H)** currently involving the left frontal, parietal, temporal, and occipital lobes.

A 66-year-old, right-handed, Chinese man came to the Neurology Unit of a local hospital in Guangzhou, China, for evaluation of a 5-day history of right-side weakness, right upper limb stiffness, and unsteady gait. An initial diagnosis of ischemic stroke was done following a magnetic resonance imaging (MRI), showing a cortical hyperintensity in the diffusion-weighted imaging (DWI) with a mild decrease in apparent diffusion coefficient (ADC) in the left occipital lobe (Figures 1A–D). The patient received treatment with lipid-lowering and anti-platelet agents.

The patient did not experience any motor improvement and developed rapidly progressive cognitive decline with dull affect, apathy, mutism with spontaneous speech severely decreased, and social withdrawal. A neurological examination 15 days later showed signs of parkinsonism with hypomimia, muscle rigidity (mostly on the right upper limb), stooped posture, and postural instability with moderate retropulsion. The patient could not walk without assistance owing to parkinsonism and gait ataxia. There was no fever, sleep disorder, obstructive apnea, gaze palsy, seizures, dysphagia, dysarthria, or upper motor neuron signs. No myoclonus or other movement disorders were observed.

**Figure 2:**
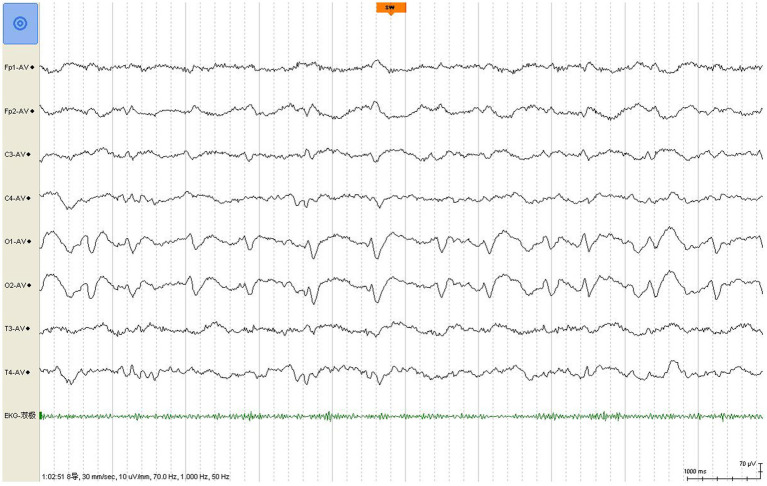
EEG shows classic periodic triphasic waves with a slow background.

**Figure 3:**
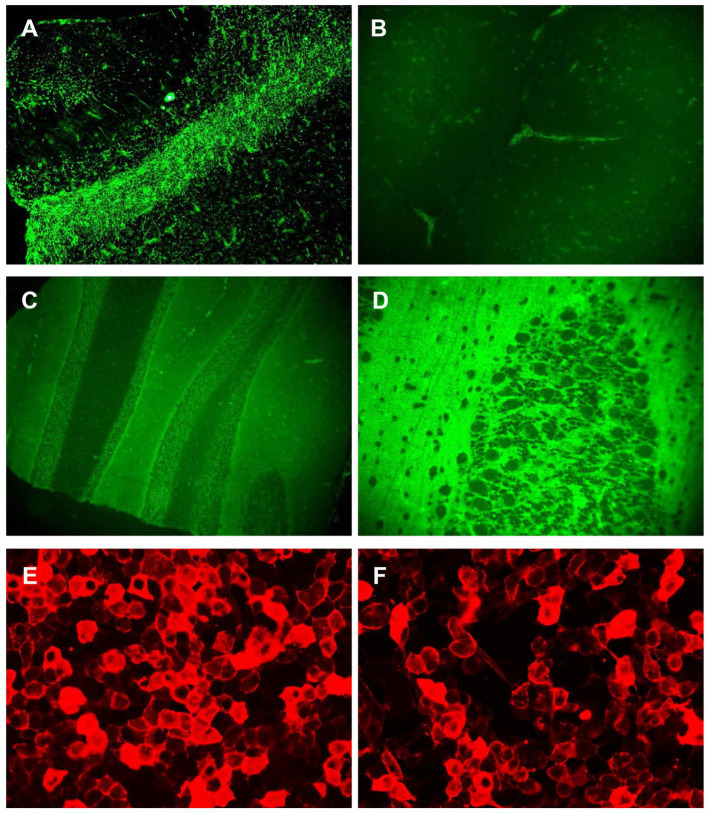
Antibody detection in serum and CSF. The result of TBA showed an uncertain neuronal surface antibody. **(A)** The immunofluorescence signal was detected in positive control (titer 1:1); **(B)** the immunofluorescence signal could not be detected in serum from the patient; **(C)** the immunofluorescence signal was detected in positive control (titer 1:50); **(D)** the immunofluorescence signal was detected in CSF from the patient; and **(E,F)** the results of CBA showed antibodies against IgLON5 were detected in both the serum (titer 1:300) **(E)** and CSF (titer 1:100) **(F)**.

A review of the initial MRI was consistent with “cortical ribboning” regarded as a typical imaging feature of sCJD (Figures 1A–D). In a second MRI carried out 2 weeks later, the cortical hyperintensities in DWI became more extensive, involving the left frontal, parietal, temporal, and occipital lobes with mild cortical restriction in the right hemisphere (Figures 1E,F), along with decreased ADC over the corresponding cortex (Figures 1G,H). The EEG showed periodic sharp wave complexes with triphasic waves (TWs), mostly in the occipital lobes with diffuse background slowing (Figure 2). Lumbar puncture (LP) showed normal CSF opening pressure (95 mmH_2_O), normal cell count, and mildly elevated protein level (55.7 mg/dL), and an analysis for 14–3-3 protein was positive (Kindstar Global, Beijing, China). Genetic sequencing for the PRNP gene by PCR and Sanger sequencing were negative (Guangzhou Kingmed Center for Clinical Laboratory, Guangzhou, China). We did not carry out the RT-QuIC test. Determination of oligoclonal bands (OCBs) showed an identical pattern in the serum and CSF (type 4 OCB pattern), indicating a possible disruption of the blood–brain barrier. Interestingly, the results of tissue-based assays (TBAs) showed the presence of an uncertain neuronal surface antibody. Compared with a positive control (titer 1:1) (Figure 3A), no immunofluorescence was detected in serum (Figure 3B); however, compared with a positive control in the CSF (titer 1:50) (Figure 3C), an immunofluorescence signal was also detected in the CSF (titer 1:50) (Figure 3D).

Then, cell-based assays (CBAs) were performed showing IgLON5 antibodies in the serum (titer 1:300) (Figure 3E) and the CSF (titer 1:100) (Figure 3F), while other autoantibodies remained negative. The Abs against the following antigens were tested: NMDAR, AMPA1, AMPA2, LGI1, CASPR2, GABAB, DPPX, GlyR1, DRD2, GAD65, mGluR5, mGluR1, neurexin-3α, GABAA, KLHL11, AChR, AQP4, MOG, GFAP, titin, recoverin, PKCγ, Zic4, Tr (DNER), SOX1, Ma2, Ma1, amphiphysin, CV2, Ri, Yo, and Hu (Methods: CBA and immunoblotting.) Additionally, human leukocyte antigen (HLA) typing showed that our patient had haplotypes DQB1*0501 and DRB1*1001, which are strongly associated with anti-IgLON5 disease (4).

The clinical presentation, MRI, EEG, and CSF results were consistent with “probable” sCJD according to the 2017 diagnostic criteria (5). However, the presence of an anti-IgLON5 Abs prompts us to consider immunosuppressive therapy. However, no signs of focal infection or systemic inflammation were observed in routine hematic, CSF, and urine tests and x-ray images. We initiated treatment with intravenous immunoglobulin (IVIg) (0.4 g/kg per day for 5 consecutive days) simultaneously with prednisolone 500 mg I.V. per day for 5 days, followed by daily prednisone 60 mg orally. However, the symptoms did not improve in the following 10 days during hospitalization. We then administered cyclophosphamide (600 mg I.V. once, followed by a maintaining oral dose of 100 mg per day) before he was discharged. Unfortunately, the patient died suddenly 3 days after being discharged home. No specific reason for his death was registered. An autopsy was not authorized by the patient’s family due to cultural reasons.

## Discussion

We present the case of a man with clinical, CSF, and neuroimaging features consistent with probable sCJD (6). The patient also had positive serum and CSF Abs for IgLON5 and haplotype frequently reported in patients with this autoimmune encephalitis (4); however, the patient did not show any characteristic clinical feature of IgLON5 encephalitis such as supranuclear gaze palsy, chorea, or sleep disorder. A previously reported patient with probable sCJD and positive IgLON5 Abs in serum had a prominent sleep disorder, supporting a possible contribution of these Abs to the clinical manifestations (7). Positive IgLON5 Abs were reported in 3 out of 920 patients with dementia, highlighting the mimetics of IgLON5-related autoimmunity with other neurodegenerative disorders (8).

The relationship between CJD and AE is complex as both conditions not only can overlap, coexisting in the same patient, but also mimic each other. Some patients with clinical pictures suggesting CJD were eventually diagnosed with AE (Table 1). Anti-voltage gated potassium channel (VGKC) complex and anti-N-methyl-D-aspartate receptor (NMDAR) Abs are the most commonly reported (9, 10). In such cases, a clinical response to immunotherapy supported the diagnosis of AE (11). In other instances, CJD has followed a well-established diagnosis of AE with characteristic facio-brachial dystonic seizures and positive leucine-rich glioma-inactivated 1 (LGI1) Abs (12, 13). However, some patients have shown positive NSA Abs in pathological confirmed CJD (Table 1) (14–17). Such a relationship was reported in a patient with neuropathology-confirmed Gerstmann–Straüssler–Scheinker and positive VGKC-complex Abs (18).

**Table 1:** Summary of reported case series with positive autoimmune antibodies in patients with suspected Creutzfeldt–Jakob disease.

Author and year	Study type	N of cases with CJD	N of cases with AE or Abs	Reported AE or antibodies	Response to immunotherapy
Geschwind et al. (19)	Observational, prospective case series	–	15	VGKC complex	92% improved
Chitravas et al. (20)	Autopsy examination	304 with a clinical diagnosis of CJD	26 (8.6%)	PACN (7), ADEM (6), LE (6), NS (4), PCD (2), WG (1)	N/A
Grau-Rivera et al. (26)	Prospective clinical case series	346 suspected CJD, 49 definite CJD	6/346 (1.7%) 0/49	CASPR (1), LGI1 (1), NMDAR (1), AQP4 (1), Tr (1), and unknown antigen (1)	100% improved or stabilized
Maat et al. (21)	Autopsy examination	384 with clinically suspected CJD	22 (6%)	Hu (1), NMDAR (1), GABA_B_ (1), CASPR2 (1), and unknown antigen (2)	N/A
Rossi et al. (22)	256 Clinical cases with probable or definite sCJD in the United Kingdom	150 had sera for Abs assessment	4 (2.7%)	VGKC-complex (2, 1.7%), NMDAR (2, 2.6%), and GlyR (1, low positivity)*	N/A
		82 sera retrospectively analyzed	4 (4.9%)	VGKC-complex (1, 1.2%), NMDAR (1, 1.2%), GlyR (2, 2.4%), and CASPR2 (3, 3.6%)*	N/A
Kerner et al. (23)	Pathological specimens at prion surveillance centers in the United States	7,934 (2,998, 38% negative for PrP)	14 (0.5%) among non-prion cases	N/A	N/A

^*^Antibodies (Abs) overlap in the same patients. ADEM, acute disseminated encephalomyelitis; AQP4, aquaporine-4; CASPR2, contactin-associated protein-like 2; sCJD, sporadic Creutzfeldt–Jakob disease; GlyR, glycine receptor; LE, limbic encephalitis; LGI1, leucine-rich glioma-inactivated 1; NMDAR, N-methyl-D-aspartate receptor; NS, neurosarcoidosis; PACN, primary angiitis of the central nervous system; PCD, paraneoplastic cerebellar degeneration; PrP, prion protein; Tr, (DNER[δ/notch-like epidermal growth factor-related receptor]); WG, Wegener granulomatosis.

Between 0.5 and 8.6% of patients with a suspected diagnosis of CJD have been reported with positive NSA Abs or pathological features of an autoimmune inflammatory disorder (Table 1) (19–23). However, some CJD patients show low titers of NSA Abs, making their pathological contribution uncertain (22). These Abs may represent a false-positive result, particularly with low titers. The possibility of a false-positive result may decrease when they are detected in the serum and CSF or in high titers. However, high titers of Abs with unclear pathogenic significance have been reported in CJD (24). As patients with CJD and AE share certain clinical features such as a rapidly progressive disorder with cognitive and behavioral problems, misdiagnoses are possible. Comparisons between patients with CJD and AE showed that seizures, hyponatremia, and dysautonomia are more commonly observed in patients with AE, whereas myoclonus, akinetic mutism, and visual disturbances are more typically observed in patients with CJD (25). It has been suggested that NSA Abs should be sought in patients with atypical features of CJD, whereas the frequency of these Abs seems lower in patients with a diagnosis of definite CJD (26). It is possible that NSA Abs represent an epiphenomenon in patients with CJD owing to the extensive cell death and antigen release. The latter phenomenon is suspected to occur in our patient as no distinct clinical features of IgLON5 were observed and no improvement with immunotherapy was achieved. However, in many instances, it is practically impossible to distinguish between true CJD and AE; therefore, a clinical response following a trial of immunotherapy can provide a strong argument in favor of AE. In this regard, patients with Hashimoto encephalitis (HE), an ill-defined inflammatory disorder, may be confused with CJD as both conditions share clinical features such as myoclonus, dementia, or cognitive impairment (27). However, patients suffering from HE have a robust response to corticosteroids (27).

The diagnosis of IgLON5 encephalitis is done by detecting specific Abs against IgLON5 in the CSF and serum by immunohistochemistry. No specific cutoff values have been established as positivity of these Abs is not expected in the general population. The relatively low titers detected in our patient may be explained by the early stage of the disease as an Abs titer is expected to increase as the disease progresses (28, 29). There are also significant associations between certain haplotypes and anti-IgLON5 disease, indicating a specific genetic susceptibility to the disease. HLA-DRB1*10:01 and HLA-DQB1*05:01 are strongly associated with the presence of IgLON5 antibodies, and the DRB1*10:01 allele was found to be 36 times more prevalent in IgLON5 encephalitis than in the general population (30). Distinctive neuropathological findings are paramount when establishing a definite diagnosis of anti-IgLON5 disease (31). Hyperphosphorylated tau deposits are the most typical finding obtained through brain biopsy, especially in the brainstem tegmentum and the hypothalamus, alongside neuronal loss. This finding contrasts with patients with sCJD, where tau deposits seem to resemble Alzheimer’s disease (AD) pathology, where deposits of neurofibrillary tangles and neuropil threads in the transentorhinal cortex precede limbic and diffuse cortical involvement, according to Braak and Braak stages (32).

The pathogenic relationship between CJD and IgLOn5 is currently unknown. Patients with CJD have been reported with increased coexistence of AD pathology consisting of aggregates of amyloid-beta (AB) peptide and hyperphosphorylated tau (p-tau) protein forming neurofibrillary tangles, neuropil threads, and abnormal neurites (33). This association has led to the hypothesis of a potential cross-seeding between prion protein, AB, and tau protein (2). However, large pathology studies have concluded that CJD and AD represent independent disease processes even if they coexist in the same brain (34). On the other hand, IgLON5 Abs have been associated with the accumulation of hyperphosphorylated tau protein in the hypothalamus and brainstem tegmentum, although this is not a universal finding in all cases with pathology samples (35). The origin of this finding is unclear; thus, it has been attributed to the intense inflammatory response caused by IgLON5 Abs. Furthermore, patients with IgLON5 autoimmunity have been reported with a high prevalence (80%) of homozygosity to microtubule-associated protein (MAPT) H1/H1 genotype, which is highly prevalent in tauopathies (30). More recently, a study using immunoprecipitation and mass spectrometry showed that IgLON5 Abs co-precipitates the IgLON family and membrane proteins, including KIDINS220 (36). The latter regulates the activity of actin components and microtubule protein resulting in the disruption of the cytoskeleton, causing dystrophic neurites and axonal swelling (37). It can be hypothesized that the coexistence of CJD and IgLON5 Abs could intensify tau pathology, magnifying neurodegeneration. However, this hypothesis is speculative, particularly considering the lack of pathological specimens in our case, and it will need further pathological confirmation.

## Conclusion

There is an overlap between CJD and AE with positive NSA Abs. In this regard, IgLON5 Abs are uncommonly observed in patients with sporadic CJD. The presence of such Abs may result from extensive neuronal destruction in an individual with a certain HLA haplotype. As deposits of p-tau protein may be observed in both disorders, a synergistic effect is possible.

## Data availability statement

The original contributions presented in the study are included in the article/supplementary material, further inquiries can be directed to the corresponding author.

## Ethics statement

Ethical review and approval was not required for this study in accordance with the local legislation. Written informed consent to participate in this study was provided by the patients’ legal guardian/next of kin. Written informed consent was obtained from the patients’ legal guardian/next of kin for the publication of this case report.

## Author contributions

XL: Data curation, Software, Visualization, Writing – original draft, Writing – review & editing. YC: Data curation, Software, Writing – original draft. LZ: Data curation, Software, Writing – original draft. WZ: Data curation, Software, Supervision, Writing – original draft. BL: Data curation, Software, Writing – original draft. JB-C: Conceptualization, Investigation, Writing – review & editing. XS: Conceptualization, Investigation, Project administration, Supervision, Writing – review & editing, Writing – original draft.
